# Maxwell-Wagner-Sillars Dynamics and Enhanced Radio-Frequency Elastomechanical Susceptibility in PNIPAm Hydrogel-KF-doped Barium Titanate Nanoparticle Composites

**DOI:** 10.1186/s11671-019-3171-z

**Published:** 2019-12-19

**Authors:** Ezekiel Walker, Yukikuni Akishige, Tong Cai, James Roberts, Nigel Shepherd, Shijie Wu, Zhiming Wang, Arup Neogi

**Affiliations:** 1 Institute of Fundamental and Frontier Sciences, University of Electronic Science and Technology of China, Chengdu, 610054 People’s Republic of China; 2 Department of Physics, University of North Texas, 210 Avenue A, Denton, USA; 3 Faculty of Education, Shimane University, Matsue, Shimane Japan; 4 Department of Materials Science and Engineering, University of North Texas, Discovery Park E118, Denton, USA

**Keywords:** High-k nanoparticles, PNIPAm, Maxwell-Wagner-Sillars, Hydrogel, Dielectric, Radio frequency, Permittivity

## Abstract

Maxwell-Wagner-Sillars (MWS) dynamics and electromagnetic radio-frequency (RF) actuation of the volumetric phase change are investigated in a hybrid polymer composite consisting of hydrogel suspended with high-k nanoparticles. Poly(N-isopropylacrylamide) (PNIPAm) hydrogels were combined with 10% KF-doped barium titanate (Ba_0.9_ K_0.1_ TiO_2.9_F_0.1_, KBT) nanoparticles with highly anisotropic dielectric properties using poly(vinyl alcohol) (PVA) to form a nanoparticle-hydrogel composite. Whereas the addition of PVA to the synthesis maintains a strong volumetric phase transition with polarization and relaxation features similar to standard bulk PNIPAm, the addition of KBT nanoparticles results in reduced volumetric phase transition and MWS polarization due to charge screening of intramolecular interactions. The added nanoparticles and modified synthesis process enhanced the dielectric permittivity of bulk PNIPAm, increased RF conductivity up to 7×, and decreased the specific heat while still maintaining a discontinuous volumetric phase transition. An RF antenna emitting at 544 kHz was only able to actuate a phase change in the composites with modified synthesis versus bulk PNIPAm. Measured heating rates were 3× greater than that of un-modified PNIPAm.

## Introduction

The use of external stimuli in hydrogel based polymers to control their physical properties, especially their thermal properties, has been a topic of immense interest in the optoelectronic [ 1], biomedical [ 2], and materials [ 3] industries. Poly-N-isopropylacrylamide (PNIPAm or PNIPA) hydrogels are polymers that are of great interest because of their ability to undergo a reversible volumetric phase transition [ 4–7]. Because PNIPAm-based hydrogels can be potentially used as artificial tissue [ 8], actuators/switches [ 9], and drug delivery systems [ 4, 7], the dielectric properties and effects of external electric or electromagnetic fields are of great importance. UV-visible light modulation of PNIPAm has limited application due to the low penetration depth of the light into an optically dispersive dielectric media.

The capability of radio frequency (RF) to penetrate deeply into materials enables remote induction of the volume phase transition. RF dielectric examinations performed on multiple formulations of PNIPAm hydrogels have revealed a general similarity with water in RF dielectric permittivity, but strong variation among the dielectric loss properties [ 10–12]. The combination of PNIPAm with high-k dielectric nanoparticles could enhance the RF electromagnetic response, accelerating the RF-induced phase transition. A proposed hydrogel hybrid of PNIPAm-based polymer embedded with high-k dielectric nanoparticle has been synthesized and exhibits enhancement in the dielectric constant and conductivity. The mesoscopic properties of the composite show its viability as a new RF susceptible hydrogel system.

Applications for PNIPAm range from biomedical [13, 14] to photonic [15], as the phase transition can be induced through photo [16, 17], thermal [17], electrical [18], pH [13], or chemical [19] stimulus. Thermal PNIPAm hydrogels exhibit a discontinuous coil-globule phase transition at around 33 °C, the lower critical solution temperature (LCST). Below the LCST, bonding occurs between the aqueous solution and the polymer chains, producing a swollen, hydrophilic gel state. Above the LCST, the bonds are rearranged due to the entropy of mixing, water is expelled from the polymer network and the gel becomes shrunken and hydrophobic. Volumetric changes of more than 10× can be readily achieved as up to 90% of the liquid solution is expelled from the polymer network [20–22].

At radio frequencies, KF-doped barium titanite (Ba_0.9_K_0.1_TiO_2.9_F_0.1_, KBT) nanoparticles exhibit attractive dielectric properties [23–25]. KBT crystals and ceramics synthesized using a sol-gel process at 650 °C, and calcined in the 650–1000 °C temperature range exhibit high dielectric permittivity at room temperature with low loss. The dielectric permittivity peaks at ~ 10,000 at 47 °C and is ~ 7000 around the LCST of PNIPAm for both the ceramics and single crystals. These properties make KBT an ideal combination for PNIPAm to form a composite with greater RF response as compared with the conventional bulk hydrogel.

### Mesoscopic Properties of the Composites

Mesoscopic properties of ionic substances can be effectively probed using dielectric spectroscopy. Examination of the complex permittivity *ϵ* ∗  = *ϵ*^′^ − *iϵ*", complex conductivity *σ* ∗  = *σ*^′^ + *iσ*", and other derived factors can reveal mechanisms related to charge transport [26] and molecular structure [27] among a host of other properties [28–31]. The modification of the synthesis process for the composites in this work was aimed at maintaining the volumetric phase change in PNIPAm-based hydrogels while enhancing dielectric responsiveness for actuating a volumetric phase change using RF. However, polar substances examined in the frequency ranges of this work behave as heterogenous systems subject to interfacial polarization effects, including electrode polarization and Maxwell-Wagner-Sillars (MWS) polarization [32].

Whereas electrode polarization is almost exclusively due to a nanolayer of charge accumulation that affects measured impedance and reveals little about the mesoscopic properties of the substance, MWS is related to molecular relaxation mechanisms [ 33], charge diffusion [33], microdomain structures that result from polarization [33, 34], and counterion polarization with molecular chain motions [30, 33]. Generally, electrode polarization occurs most strongly in the low frequency ranges below 10 kHz or so. Its signature is generally associated with a strong increase in the real part of the permittivity, *ϵ*′, and a corresponding minimum in in *σ* ′ ′ [30].

The dielectric response of KBT suspended in PNIPAm-based hydrogel was investigated using dielectric spectroscopy. Bulk- [35], micro- [ 11], and nano- [36] forms of PNIPAm hydrogels maintain similar phase transition properties. The chemical stability of KBT in the polymer synthesis process motivated the use of bulk PNIPAm as the hydrogel of choice. In this work, a high-k dielectric doped hydrogel polymer has been realized using 10% KF-doped BaTiO_3_ nanoparticles calcined at 800 °C. The physical properties of this material, such as dielectric constant, loss, and RF conductivity for the feasibility of RF-modulation, are reported and compared with free radical polymerized PNIPAm using dielectric spectroscopy. The enhancement of the dielectric properties was estimated based on the potential for increased RF responsiveness, specifically in the 0.1–1.0 MHz frequency range. The resulting RF heating and effects of the modified synthesis process on RF susceptibility are reported and discussed below.

## Results and Discussion

### Bulk PNIPAm

Bulk poly (N-isopropylacrylamide) hydrogel formed using free radical polymerization serves as the base material for all composites in this work and is presented for reference. The dielectric characteristics signifying the onset of electrode polarization versus MWS are still a topic of investigation. For the net measured dielectric properties, recent work indicates that the onset of electrode polarization (EP) occurs when *ϵ* ′ (*f*) starts to show a saturation plateau while a simultaneous peak in *ϵ* ′  ′ (*f*) is present [ 37]. MWS, however, is indicated by an inflection point in the increase of *ϵ*^′^(*f*) which coincides with a peak in *ϵ*^′′^(*f*).

**Fig. 1:**
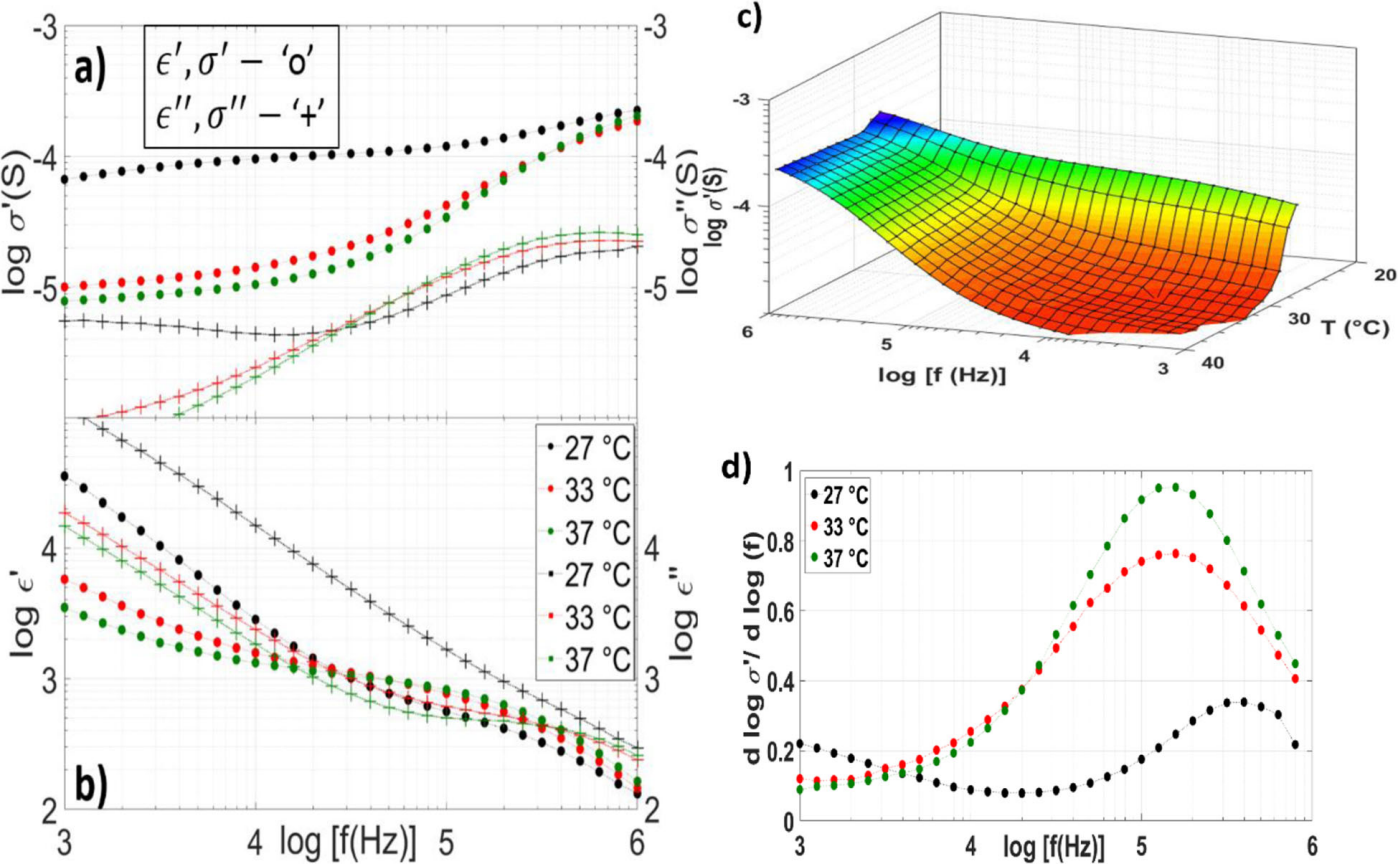
Bulk PNIPAm temperature-dependent spectroscopy. Real and imaginary conductivity (**a**, top left), dielectric constant (**b**, bottom left) at 27 °C (black), 33 °C (red), and 37 °C (green) for bulk PNIPAm formed using free radical polymerization. **c** (top right) The temperature (T)–dependent conductivity *σ* ′ (*f*, *T*) shows a strong decrease around the phase transition temperature of ~ 32 °C. **d** (bottom right) d (log *σ* ′ )/d log (f) at 27 °C (black), 33 °C (red), and 37 °C (green) showing the extent of MWS polarization in the sample

**Fig. 2:**
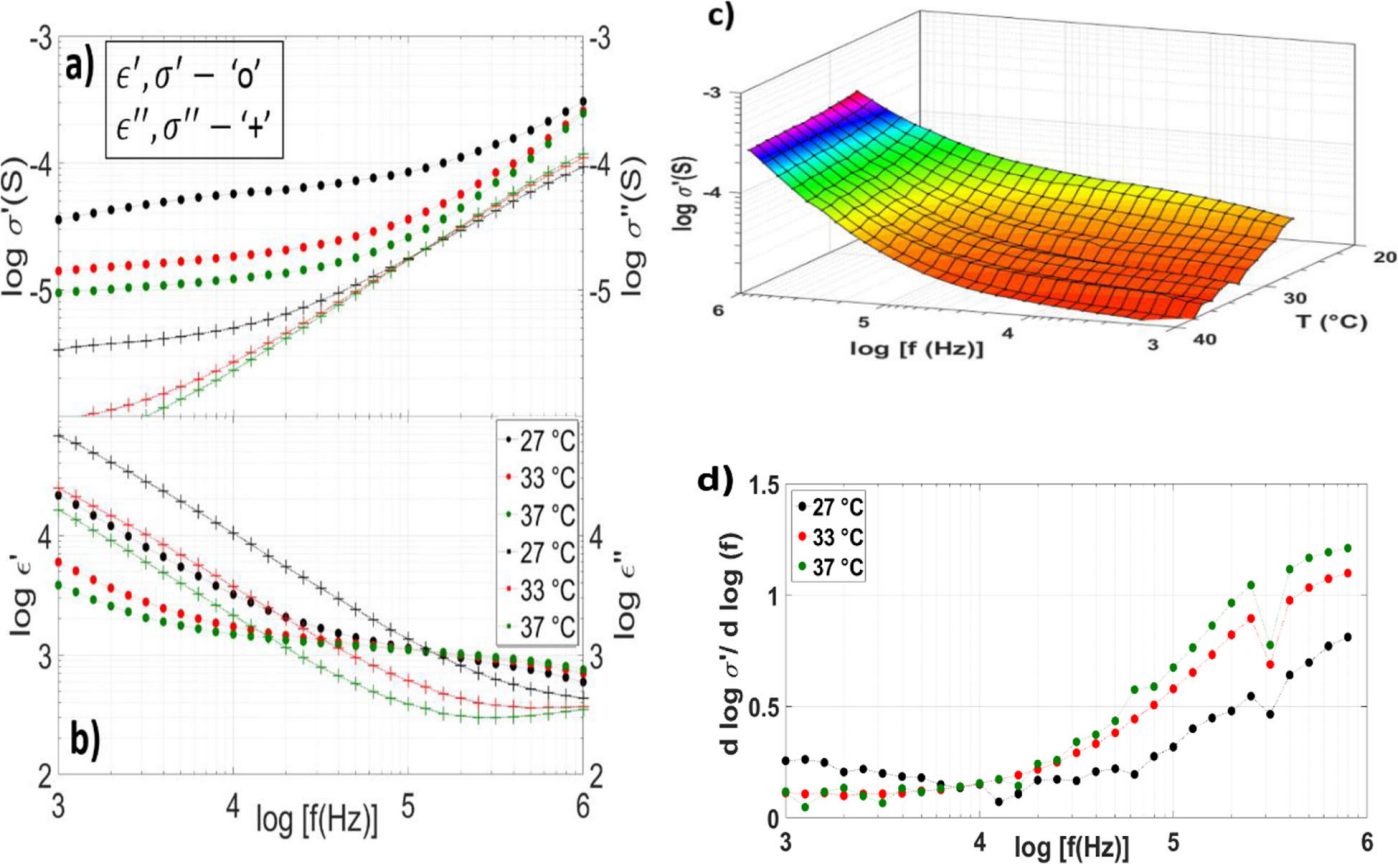
PVA-PNIPAm temperature-dependent spectroscopy. Real and imaginary conductivity (**a**, top left), dielectric constant (**b**, bottom left) at 27 °C (black), 33 °C (red), and 37 °C (green) for PVA+ PNIPAm formed using free radical polymerization. **c** (top right) The temperature (T)–dependent conductivity *σ* ′ (*f*, *T*) is passivated as compared with bulk PNIPAm, but still shows increased MWS with temperature. (d, bottom right) d (log *σ* ′ )/d log (f) at 27 °C (black), 33 °C (red), and 37 °C (green) showing the extent of MWS polarization in the sample shifted to a higher frequency as compared with bulk PNIPAm due to decreased particle spacing.

Figure 1 represents the dielectric behavior of bulk PNIPAm at 27 °C, 33 °C near the phase transition temperature, and 37 °C. It is clear from Fig. 2 that no plateaus in *ϵ*^′^(*f*) are resolved in the frequency range studied in this work for bulk PNIPAm. As the onset of EP is indicated by an *ϵ* ′ (*f*) saturation plateau, it is not a significant contributor to the dielectric behavior and MWS is the dominant contributor to observed polarization effects in this work. Figure 1 a and c show a strong decrease in conductivity at lower frequencies due to MWS as observed in other works [27]. PNIPAm is a heterogeneous system due to free and bound water molecules. N-Isopropylacrylmide polymer chains, and other impurities and multiple relaxations appear in the shoulder formed around 100 kHz in *ϵ*′ and *ϵ*^′′^. The $\frac{d\log \left({\sigma}^{\prime}\right)}{d\ \log (f)}$ of bulk PNIPAm shows the extent of MWS polarization, with the onset indicated by a minimum in *ϵ* ′ ′ [27]. Figure 1 a and d show good agreement with other literature as the onset of MWS shifts to a lower frequency above the LCST, and increases in intensity once the hydrogel transitions from the coil to globule phase. Domain ordering is the macroscopic ordered or semi-ordered arrangement of charges in a system composed of polar particles and manifests in the fractional shape parameters of the relaxation spectra of *ϵ* ′  ′ (*f*) 28, 33, 38, 39. Generally, for a relaxation peak, a flatter slope on the low frequency side is associated with molecules behaving as a lattice with greater domain order, and a slope approaching − 1 on the high frequency side is associated with molecular clusters behaving as a highly correlated singular entity 33. While the extent of MWS polarization is deduced from Fig. 1d, a, thorough analysis of domain order in the hydrogel system is left for other works.

### PVA-Modified Bulk PNIPAm

Poly(vinyl alcohol) (PVA) has been shown to be immiscible due to very weak interaction with polymeric pairs in PNIPAm [40]. As the volumetric phase transition is a function of the bonding characteristics of NIPA chains with water, PVA as an additive is ideal if the goal is to maintain the coil-to-globule phase transition that is attractive for many applications. As discussed in the “Methods” section, PVA was added to standard bulk PNIPAm monomer to increase monomer viscosity and prevent the precipitation of KF-doped BaTiO_3_ nanoparticles to be added later in the process. Visual verification of the coil-globule phase transition properties is given later in this work. From the dielectric spectra in Fig. 2, there is modification in the contribution of MWS polarization, but the general behavior does not deviate significantly from bulk PNIPAm. For this text, except where explicitly stated, PVA refers to PVA + PNIPAm.

As in the case of PNIPAm, though the characteristics of interfacial polarization are clearly represented in both *σ*^∗^ and *ϵ*^∗^, no plateaus are observed in the *ϵ*′ (Fig. 2a,b). Therefore, the contributions of electrode polarization can be neglected. The contour of *σ* ′ (*f*, *T*) is a function of MWS polarization. In bulk PNIPAm, the discontinuous coil-globule phase transition is well represented in the *σ* ′ (*f*, *T*) spectrum (Fig. 1c). The discontinuity is passivated in PVA likely due to residual hydrophilic poly(vinyl alcohol) chains that were not diluted from the hydrogel during synthesis (Fig. 2c). MWS is still increased above the LCST, but shifted to a higher frequency (Fig. 2d). For a heterogenous system, the shift of MWS to higher frequencies is due to increased particle concentration which is to be expected with the additional poly(vinyl alcohol )[27].

### KF-BaTiO_3_ Nanoparticle Dispersed Bulk PNIPAm

**Fig. 3:**
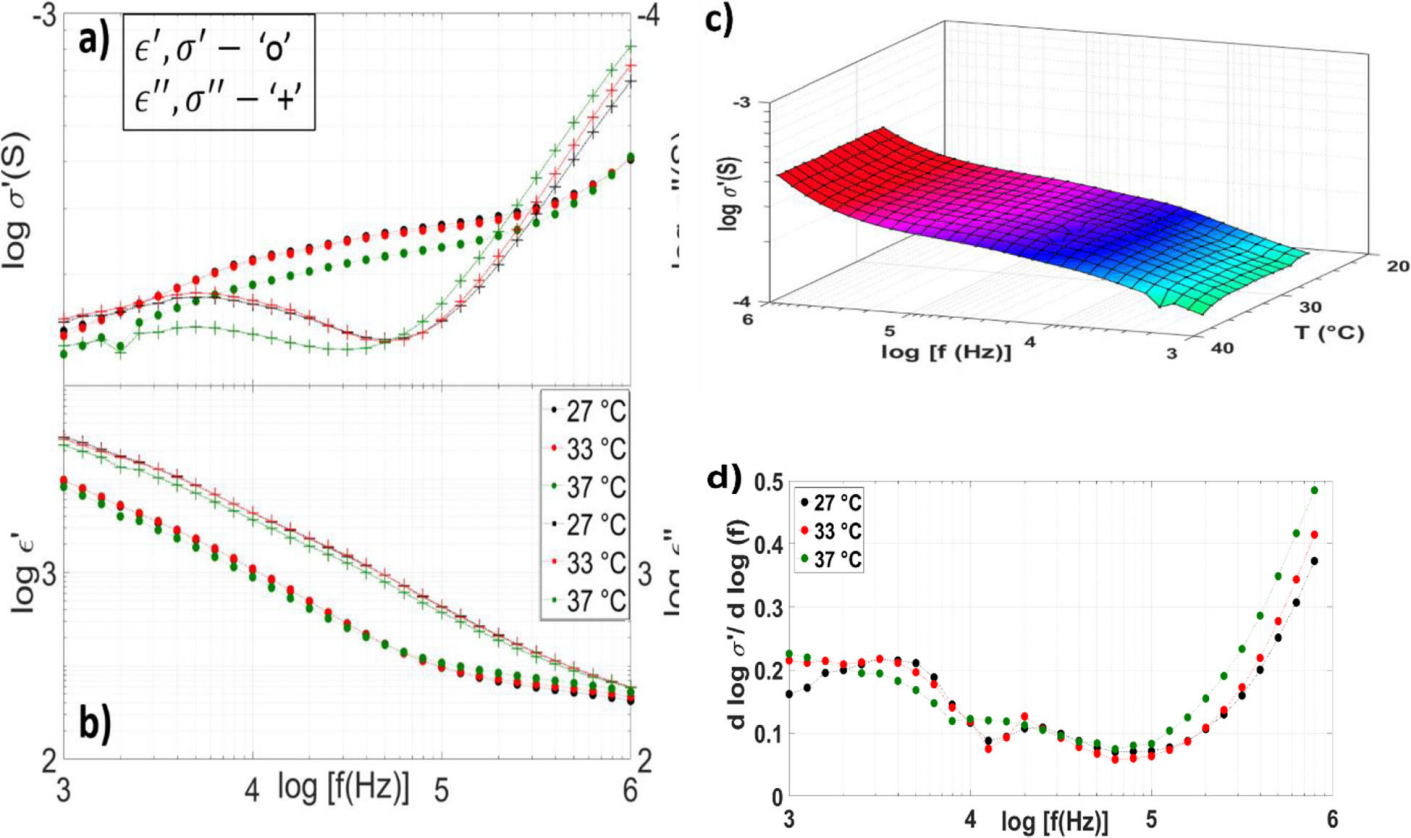
KBT-PVA-PNIPAm temperature-dependent spectroscopy. Real and imaginary conductivity (top left), dielectric constant (bottom left), electric modulus (right) at 27 °C (black), 33 °C (red), and 37 °C (green) of bulk PNIPAm with dispersed KF-BaTiO_3_ nanoparticles.

High-k KF-doped BaTiO_3_ (KBT) nanoparticles were dispersed in bulk PNIPAm to enhance the dielectric properties for eventual RF actuation. Actuation is examined in the following sections. However, though all samples maintained the volumetric phase transition associated with PNIPAm, the KBT+PVA+PNIPAm composites demonstrated the least visual coil-globule change. These results are also apparent in the dielectric spectra which significantly deviate from that of PVA or bulk PNIPAm. For brevity, KBT refers to KBT+PVA+PNIPAm hydrogel composite unless otherwise stated in the remainder of this work. The minima in the *σ* ′  ′ (*f*) spectra at ~ 50 kHz coinciding with an inflection point in *ϵ* ′ (*f*) is a feature of MWS (Fig. 3a, b). Unlike PVA and PNIPAm, interfacial polarization does not undergo a significant increase above the LCST. This is evidenced by the lack of a significant change in the dispersion characteristics of either *σ*^∗^ or *ϵ*^∗^with increasing temperature (Fig. 3a, b, c). Even less of the change in *σ*′ with frequency is resolved in Fig. 3d as compared with PVA and PNIPAm composites. Regardless, it is apparent that relatively little change follows the coil and globule transition.

Conversely, the addition of KBT nanoparticles to the composite increased both *σ*^∗^ and *ϵ*^∗^ as was the initial motivation for the work. Qualitatively, the addition of KBT also passivated dynamic domain ordering the temperature-dependent dispersion curve of both the real and imaginary components of *ϵ*′. As with PNIPAm, a thorough investigation into domain ordering in this complex system is left for future works. However, passivation of both the phase transition and domain ordering may result from KBT nanoparticles screening the charge interactions between local NIPA chain, NIPA chain clusters, and water.

### RF Actuation

**Fig. 4:**
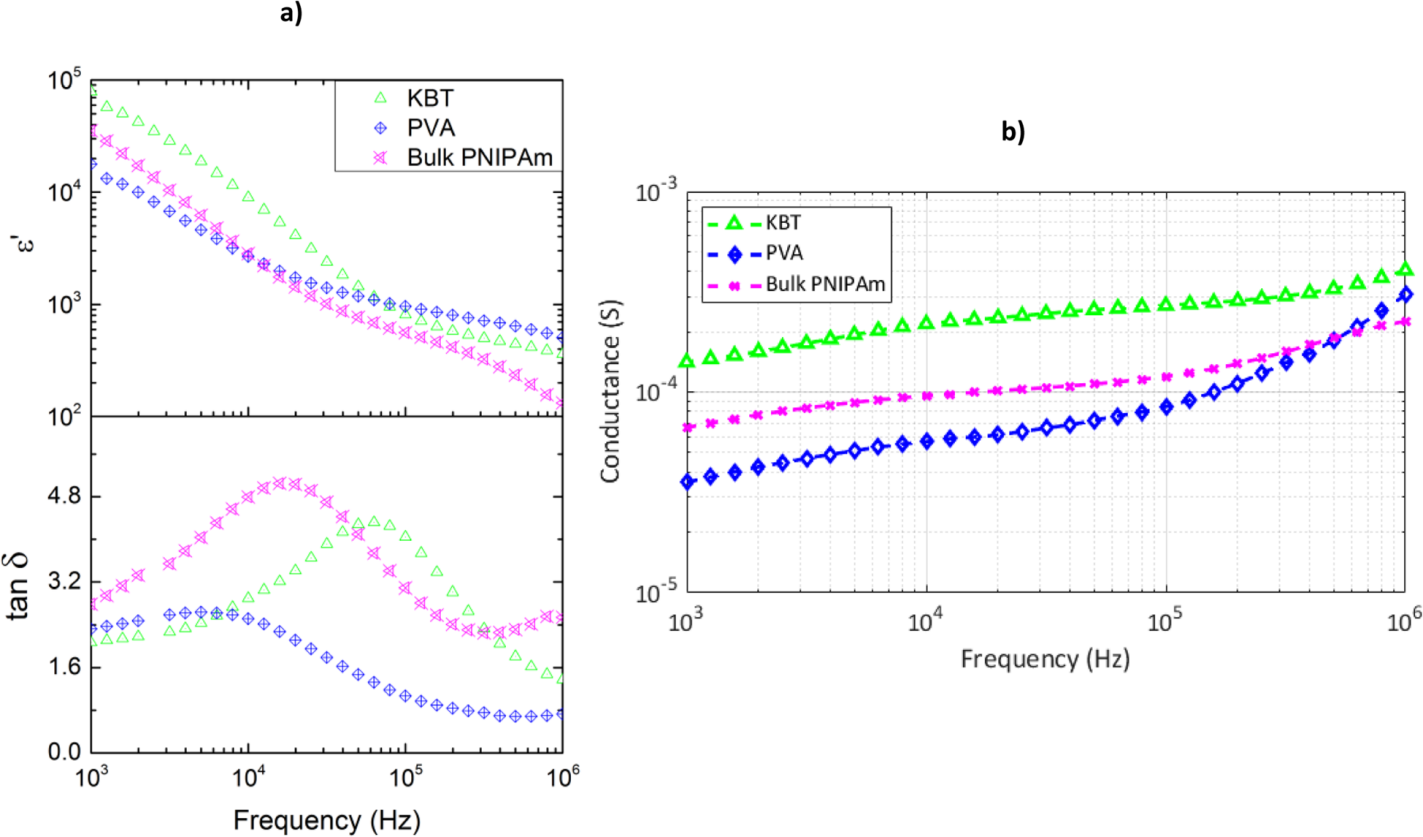
Direct comparison of PNIPAm, PVA, and KBT composites. **a** Dielectric constant (ε′) and loss tangent (tan δ) of hydrogels in RF frequency range at 27 °C. Characteristics of polarization contributions to the dielectric measurements begin at ~ 100 kHz for KBT800, ~ 20 kHz for PNIPAm, and ~ 10 kHz for PVA. **b** Conductance of KBT, PVA, and Bulk PNIPAm at 27 °C

The additions of PVA and KBT nanoparticles to the free radical polymerization process increases ε′ of both composites compared with PNIPAm despite contributions from surface polarization effects [28, 41]. At 544 kHz, the relative permittivity increases from 206.53 (PNIPAm) to 425.21 (KBT) and 612.95 (PVA) with an error of < 5% for each calculated value (Fig. 4a). The addition of poly(vinyl alcohol) has shown the ability to increase dielectric permittivity in aqueous systems due to it hydrophilic effects [42]. The onset of Maxwell-Wagner Sillars (MWS) and electrode surface polarization effects are indicated by the change in slope in ε′ and relaxation peaks in tan δ in Fig. 4a. The combination of the earlier onset characteristics, and increase in conductivity in Fig. 4b show that the addition of KF-doped BaTiO_3_ nanoparticles increases ionicity in the hydrogel composite. Increasing the conductivity while maintaining discontinuous elastic properties makes added KBT a potential option for electrically triggered polymer based actuators, muscles, or tissues. The dielectric constant (ε′) and loss tangent (tan δ), the frequency of actuation, and the transient thermal properties of the composites are the critical components for RF to stimulate a volumetric phase change in hydrogel. The frequency dependence of the penetration depth and energy deposition required in the samples studied indicate that the frequency range of 100 kHz–1.0 MHz would be effective to induce a volumetric phase change.

**Fig. 5:**
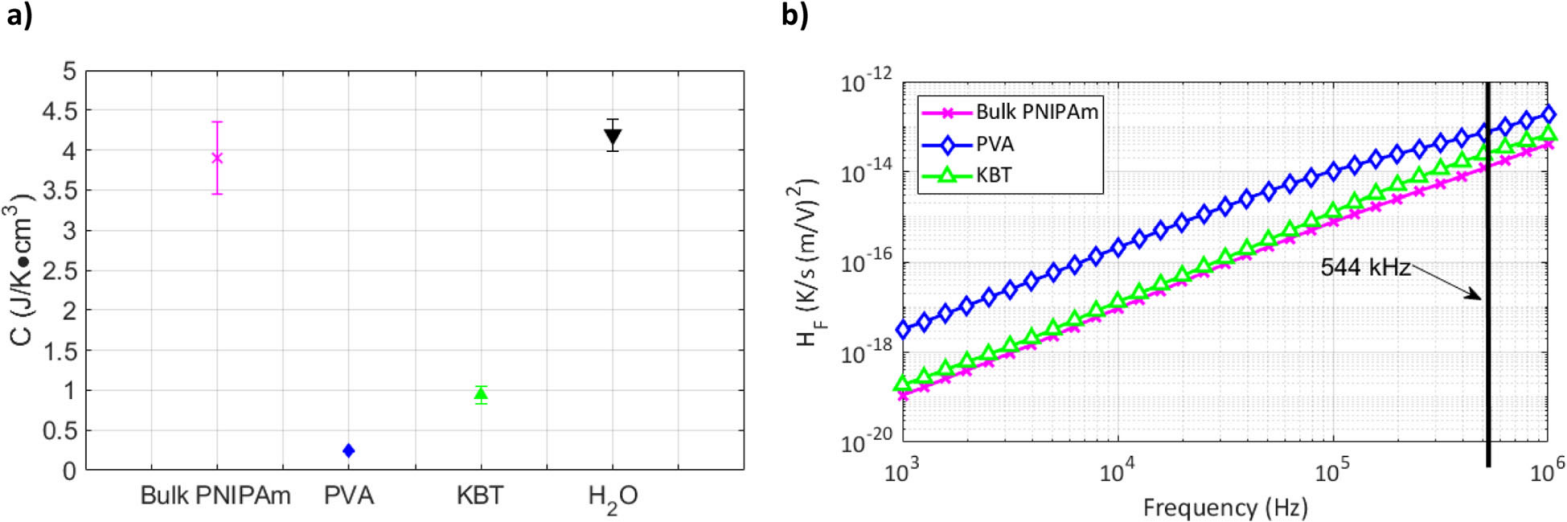
Specific heat and RF heat factor. **a** Volume-specific heat capacity (C) for bulk PNIPAm, PVA-PNIPAm hydrogel, KBT hydrogel, and water derived from measured specific heat and volume for each sample. (left) The addition of PVA to the monomer solution in the free-radical polymerization process lowered the specific heat from nearly 4.0 J/(g∙K) in PNIPAm to 0.25 J/(g∙K) in PVA and 0.95 J/(g∙K) in KBT. **b** The heat factor (Hf). Equation 2 sans Ea with all the measured dielectric and material parameters. 544 kHz is the RF actuation frequency in this work (right)

The volume-specific heat capacity, *C*, of each material is the other contributing factor to RF heating (Fig. 5a). Preliminary models assumed specific heat properties that would be similar to bulk PNIPAm. However, the lack of agreement between the measured data and the model led to additional calorimetry to determine the effective specific heat of the composites. PNIPAm hydrogels comprise a complex system composed of 90+ wt. % water with poly(N-isopropylacrylamide) polymer chains with groups that form hydrogen bonds with water based on temperature conditions. For the KBT and PVA samples, poly(vinyl alcohol) will not bond with PNIPAm chains, but is hydrophilic and may form hydrogen bonds with water [43]. Strong intermolecular interactions produced by hydrogen bonding have been shown to increase thermal conductivity [43]. In this case, the addition of PVA to the synthesis process increases the overall hydrogen bonding capability of the composite without disrupting PNIPAm ability to interact with ambient water. The addition of poly(vinyl alcohol) to the PNIPAm hydrogel synthetization process significantly lowers *C*_*p*_ from 3.70 $\frac{J}{g\bullet K}$ in bulk PNIPAm to 0.25 $\frac{J}{g\bullet K}$ in PVA hydrogels and 0.95 $\frac{J}{g\bullet K}$ in KBT hydrogels as derived from measurements and Eq. 1. In this work, the density of PNIPAm in the hydrophilic state was measured to be 1.06 g/cm^3^. PVA and KBT densities were measured to be 0.94 g/cm^3^ and 0.99 g/cm^3^ respectively. The combination of the two factors results in a significantly lower *C* that dominates the contribution to heating for the PVA and KBT samples (Fig. 5). For Fig. 5b, *H*_*F*_ is defined as the heat factor for dielectric heating, where ${H}_F=\frac{{\mathrm{D}}_{\mathrm{H}}}{C\bullet {E}_A^2}$.

**Fig. 6:**
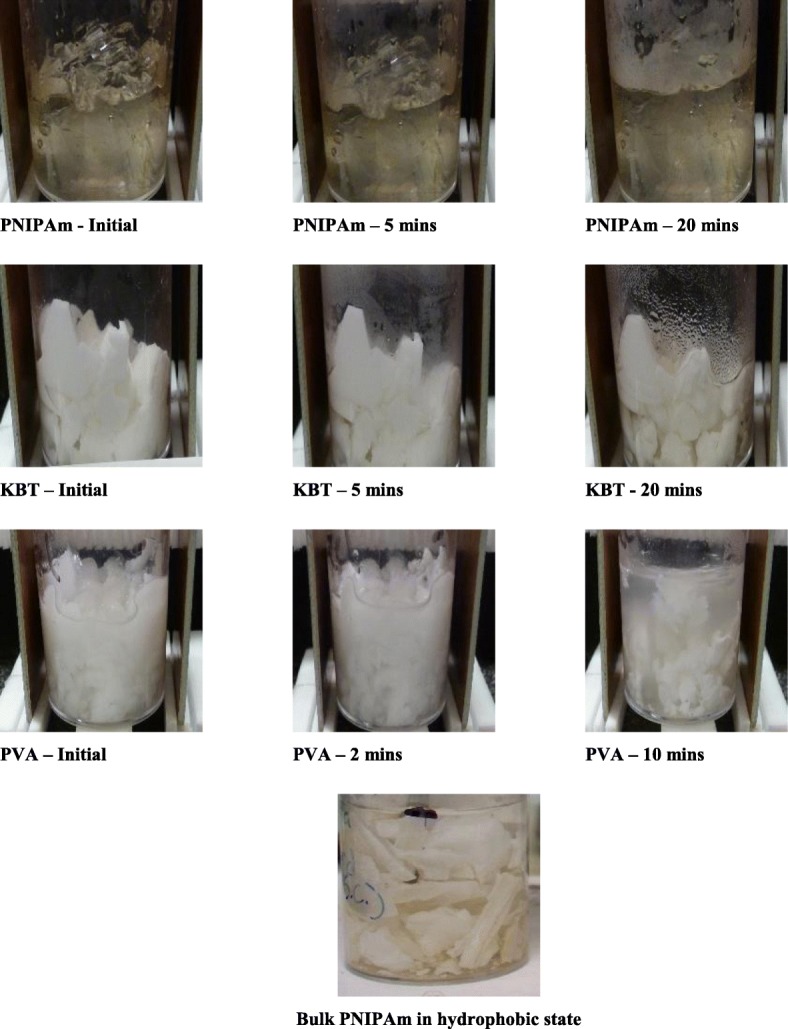
Time-dependent response of PNIPAm, PVA, and KBT to applied RF. Images of PNIPAm, KBT, and PVA hydrogels undergoing exposure to RF at 544.4 kHz in a parallel plate antenna setup. Bulk PNIPAm (top) does not show any discernible volumetric change while KBT (2nd row) and PVA (3rd row) show RF-induced volumetric changes. PVA undergoes the most extensive volumetric phase change within 10 min of application, while KBT at 20 min of application. Bulk PNIPAm after undergoing its phase change attained using conductive heating (bottom)

RF at 544 kHz was applied to each sample in a parallel plate antenna arrangement. The lower volume-specific heat capacity greatly increases the projected heating capability of the PNIPAm-based composites using non-contact RF. The methodology is given below. Figure 6 contains images of each sample after a set time exposure to RF. Each sample was exposed under the same conditions, with initial conditions being room temperature. The goal was to observe an induced volumetric phase change. All samples were exposed to RF for a maximum of 30 min. Bulk PNIPAm did not show visible change within the exposure window. Both KBT and PVA show volumetric phase changes with applied RF. In agreement with Fig. 5b, PVA starts to undergo a phase transition within 2 min of exposure, and undergoes complete collapse with 10 min of RF. KBT begins showing collapse at 5 min of applications, but does not show significant effects until 20 min of RF. The figure also gives a representative image of bulk PNIPAm after undergoing its volume phase transition induced by resistive heating.

## Conclusion

In this work, we detailed the synthesis of a new hybrid polymer with enhanced dielectric properties and phase change susceptible to RF application. Maxwell-Wagner-Sillars interfacial polarization was confirmed in each composite and in the case of PVA+PNIPAm and bulk PNIPAm, and is significantly affected by the coil-globule phase transition. Domain ordering was not thoroughly investigated in this work, but the qualitative, comparative behaviors of *ϵ*^∗^and *σ*^∗^demonstrate that the addition of KBT nanoparticles reduces the dynamics in MWS-related domain ordering likely due to charge screening of NIPA-NIPA, NIPA-water, and unbound water intramolecular interactions. The addition of KBT also reduces the strength of the coil-globule transition.

The radio-frequency active polymer composites functionalized with high-k dielectric nanoparticles have enhanced dielectric properties while maintaining relatively weak volumetric phase change abilities. The increase in ε′ does not, however, result in superior RF heating characteristics. The addition of poly(vinyl alcohol) to the synthesis process increases ε′ and modifies tan δ, but also greatly reduces the volume-specific heat capacity, leading to better RF heating susceptibility. Poly(vinyl alcohol) does not disrupt the ability of PINPAm to undergo its discontinuous volumetric phase change, and so is shown to be an ideal candidate as an additive. For modulation using electromagnetic waves, this is a step towards making a material that can meet the practical engineering requirements for widespread application without compromising the volumetric abilities of the hydrogel. The seven-fold increase in the conductivity of KBT composites over standard PNIPAm can reduce the necessary input power needed to actuate a phase change in the gel electrically. This hybrid PNIPAm material can be modulated using RF in a non-contact mode and also or through low-energy electrical means due to enhanced electrical conductivity.

## Methods

### Synthesis of KF-BaTiO_3_ Nanoparticles

Nanoparticles of KBT were formed using a sol-gel technique [25, 44, 45]. Titanium tetraisopropoxide (Ti{OCH(CH_3_)_2_}_4_), barium diethoxide (Ba(OC_2_H_5_)_2_), and KF powders were dissolved at a molar ratio of 1.0:0.9:0.1 in sequence in a mixed solution of methanol and 2-methoxyethanol in a dry glove box with an N_2_ gas flow. Hydrolysis was carried out with distilled water by spraying the solution after being cooled to 0 °C while being magnetically stirred. The resulting gel was dried at 50 °C for 24 h, then at 90 °C for 3 days. The dried gel was then pulverized and calcined at 650 °C to remove organic matter. The final nanoparticles were formed by firing the calcined powder at 800 °C for 2 h.

**Fig. 7:**
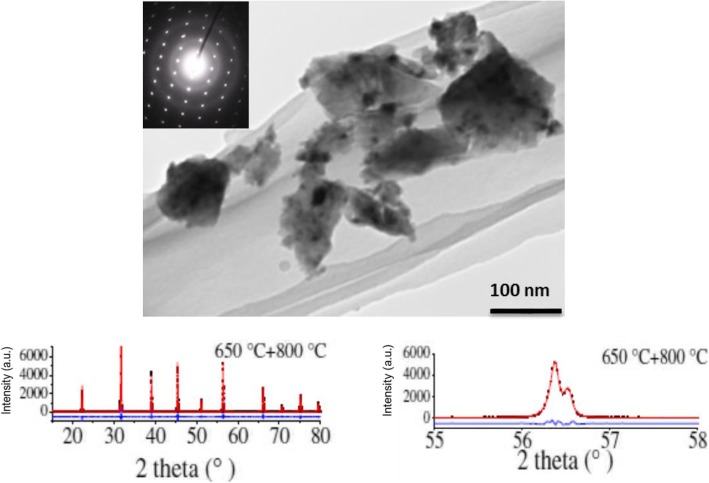
TEM, XRD, and diffraction pattern of KF-BaTiO_2._ TEM, the diffraction pattern, and XRD scan of KF-BaTiO_3_ calcined at 800 °C as performed in 25. The nanoparticles range in size from 70 to 200 nm with a lattice constant of 3.99145${\AA}$.

The crystallization and phase purity of KF_0.1_-BaTiO_3_ crystals were studied by Akishige et al. in other works [23–25]. Figure 7 shows the cubic crystalline diffraction of the particles which vary in size from 70–200 nm. While the dielectric properties of standalone nanoparticles were not investigated, dielectric characterization of ceramics formed from the powders and crushed single crystals of KBT was completed. The ceramics were prepared by the spark plasma sintering (SPS) technique: KBT650 powders were pressurized into a pellet at 20 MPa and sintered at 1000 °C for 5 min in vacuum. The single crystals were prepared by a KF- flux method: a mixture of BaCO_3_, TiO_2_, and KF was melted at 1073 °C and cooled to 976 °C for 2 h and to 796 °C for 8 h. sequentially. As the calcine temperature increases, the size of the crystal increases from ~ 70 nm at 650 °C to ~ 200 nm at 800 °C, and the crystal habit quality diminishes as F evaporates above 740 °C. Despite the variation in synthesis, both ceramics and crushed single crystals show similar dielectric behavior and increased dielectric permittivity with exposure to high temperatures with a room temperature dielectric permittivity of greater than 5000 (ε′ > 5000) in the kHz RF range. The behavior of the ceramics and crushed single crystals was used as a guide for the powder form nanoparticles. KBT exhibits RF dielectric permittivity (ε′) and loss tangent (ε″/ε′, tan δ) of ~ 10,000 and ~ 0.05 around the PNIPAm hydrogel LCST at 10 kHz. Therefore, KBT powders calcined at 800 °C were added to PNIPAm-based hydrogels to increase their RF dielectric response. The hydrogel-KBT composites refer to free-radical polymerized bulk form PNIPAm-based hydrogel with KBT powders calcined at 800 °C suspended in the polymer network.

### Hydrogel-KBT Composite Synthesis

The properties of PNIPAm in various forms have been well studied. However, KBT nanoparticles were observed to rapidly precipitate out of water and the monomer solution used in PNIPAm free-radical polymerization. An additional chemical as described below was added to the monomer solution to significantly lower the precipitation rate of the nanoparticles while maintaining the hydrogel volume phase transition. Pre-polymerized poly(vinyl-alcohol) (PVA) was used to reduce precipitation in the hydrogels because it not only increases monomer solution viscosity but also lacks the requisite vinyl groups for chemical bonding to NIPA polymer chains [40]. Though studies have shown PVA to be an excellent steric stabilizer for PNIPAm microgel particles [46], the role of PVA in this work was to increase the viscosity of the KBT-monomer solution to prevent the precipitation of the KBT nanoparticles while also maintaining the volumetric transition properties of the PNIPAm gel. Without bonding to PNIPAm, PVA can be diluted out of the hydrogel. With PVA in the monomer solution, precipitation was experimentally determined to slow from ~ 1 min to 3 days.

The hydrogel-KBT synthesis process is detailed in other works [47], and is detailed here for convenience. N-Isopropylacrylamide monomer (PNIPAm, TCI Chemicals), N, N’-Methylene-bisacrylamide crosslinker (BIS, Polysciences Inc), and DI water were mixed together in a ratio of 0.10 (wt.):0.02 (mol PNIPAm):0.84 (wt.) to make a monomer solution. Poly(vinyl alcohol) (PVA, Polysciences Inc.), at 2 wt.% of the total mixture, and KBT nanoparticles, at 1 wt.% of the total mixture, were then added to the monomer solution. The solution was heated to 50 °C and stirred for > 24 h to ensure the dissolution of the PVA into the monomer solution, and the dispersion of the nanoparticles in the solution. Dispersion of the KBT powders was achieved with magnetic stirring of the low-precipitation solution for more than 24 h. The composite solution was then set in an ice-bath, pumped with N_2_ for > 1 h to remove adsorbed oxygen while being magnetically stirred. Ammonium persulfate (APS) and tetramethylethylenediamine (TEMED) were used as the initiator and accelerator for final polymerization in the PVA PNIPAm bulk gels/composites. The final gels were immersed in deionized (DI) water for > 2 days and the water was changed every ~ 6 h to remove residual initiator and accelerator.

**Fig. 8:**
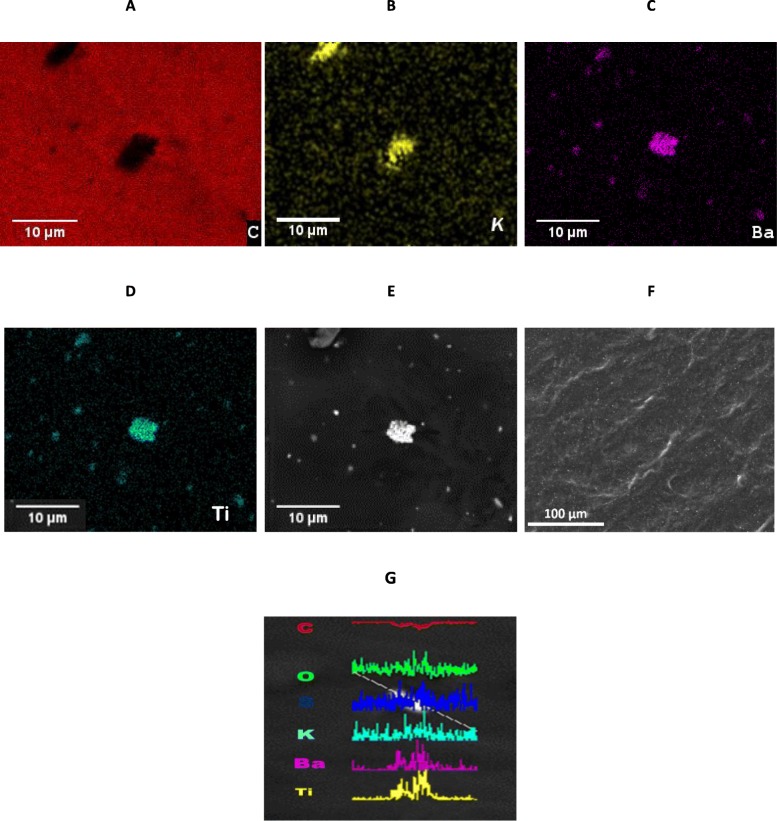
EDAX of KF-BaTiO_2_ nanoparticle-hydrogel composites. Images of KBT nanoparticles in PNIPAm-based hydrogel. **a**–**c** 25 kV EDAX compositional images of KBT in hydrogel. **a** Carbon, the indicator for the polymer. **b**, **c**, **d** K, Ba, and Ti the indicators for KF-BaTiO3. **e** Standard image without elemental filtering. **f** Dispersed KBT nanoparticles in PNIPAm hydrogels. EDAX show clustering of KBT nanoparticles, but adequately uniform dispersion of the nanoparticles/clusters throughout the hydrogel. The medium is homogeneous compared with the length of the RF wave at 0.01–1.00 MHz. **g** Elemental composition of KBT nanoparticle cluster

Pre-polymerized PVA is soluble in water, but does not readily attach or cross-link with PNIPAm polymer chains in the free radical polymerization procedure due to the lack of the necessary vinyl group needed to attach to PNIPAm. To remove excess PVA, the hydrogel was heated above the LCST to ~ 50 °C, the excess liquid was removed and replaced with DI water at 20 °C, the gel rehydrated, and the process was repeated for each sample to remove PVA. Figure 7 shows EDAX images of KBT powders dispersed in PVA-PNIPAm. Though the KBT shows clustering aggregations that vary in size up to 10 μm (Fig. 8a–d), the relatively uniform dispersion visible in Fig. 8f was used as a satisfactory validation of the dispersion technique.

In all samples in this study, the volumetric phase transition associated with PNIPAm, but not observed in strictly PVA polymers, was experimentally verified at the standard LCST temperature (~ 33 °C). The volumetric properties of the PVA-PNIPAm hydrogel maintained a discontinuous volumetric phase transition at ~ 33 °C in the same fashion as standard PNIPAm. This is to be expected as the phase transition is due to the hydrogen bonding interactions between PNIPAm and water. KBT nanoparticle-gel composites were made using the PVA PNIPAm bulk gel. The structural properties of the dielectric nanoparticles and the hydrogel are shown in Fig. 7. The use of PVA as an additive for viscosity purposes was empirically determined after significant precipitation of KBT was observed in standard free-radical polymerization bulk gel solutions. Other combined solutions did not maintain the volumetric phase change properties. The amounts of each component of the mixture were also empirically determined, and the volumetric phase change properties for the bulk composites were experimentally verified to be maintained. For the duration of this work, “PVA” is used to indicate PVA- PNIPAm hydrogel and “KBT” to indicate KBT800-PVA-PNIPAm hydrogel.

### Temperature-Dependent Dielectric Measurements

Dielectric properties were measured with the sample placed in the interior of a sealed copper faraday cup lined with Teflon. Copper electrodes 12.90 mm in diameter spaced to the thickness of the sample cell were centered in the cage. The sample cell consisted of a Teflon ring with an inner diameter of 5.0 mm, an outer diameter of 11.11 mm, and thickness of 2.8 mm with conducting copper tape attached to seal one side of the cell to hold liquids. The sample cell was fixed between the two electrodes using pressure from Teflon screws on the upper and lower portions of the electrodes. Temperature was monitored using a Teflon-tape encased K-type thermocouple with an accuracy of ± 0.05 °C placed at the base electrode. A FDC-C21 temperature controller was used to control an electrically insulated resistive heater placed inside the faraday cage ~ 3 cm away from the sample. The temperature and dielectric data was recorded at each degree from 27–39 °C ± 0.3 °C.

A Solartron 1260A Impedance Gain/Phase Spectrum Analyzer was used to measure the dielectric properties of the KBT-gel composites. Complex impedance was measured at 10 points/decade from 1–1000 kHz with an electric field strength of 500 mV rms. The dielectric constant (ε′), conductance (σ), loss tangent (tan δ), and other dielectric values of each sample were derived from the measured complex impedance and calibrated parameters of the sample cell. The samples were fully hydrated at the outset of each measurement. As hydrogels generally are > 90 wt. % water, electrode and interfacial polarization effects were expected and observed [48].

### RF-Induced Sample Phase Change

RF was applied to the samples using a parallel plate antenna setup. Pre-weighted samples were placed into capped acrylic vials that did not exhibit heating when exposed to RF. Two 60 mm × 30 mm single-sided copper plates spaced 36 mm apart using Teflon spacers acted as the antennae. A dual, anti-phase, harmonic LRC setup generated 544 kHz RF at 8500 V peak-to-peak in air. The samples were placed between the antennae with no physical conductive connection between the sample and the plates. For measuring temperature increases, the high intensity of the RF field prevented in situ temperature measurement. RF was applied for 5 min to the samples at room temperature and the temperature recorded at the beginning and end. In order to gauge the effects of RF, images of the samples were taken with active RF application at 2- and 5-min intervals for 30 min.

### Energy Dissipation, Dielectric Heating, and Heat Capacity

The derivation of energy dissipated due to an electromagnetic field in a dielectric material is given in other works [47]. For RF applied using a parallel antenna setup, the dielectric contribution to heating is

1 $$ {D}_H=\frac{2\pi {\epsilon}_0\cdot f\left[ Hz\right]\cdot \tan \delta \cdot {\left|{E}_a\left[\frac{V}{m}\right]\right|}^2}{\epsilon \hbox{'}\left(1+{\left(\tan \delta \right)}^2\right)} $$

where *E*_*a*_ is the RF electric field amplitude in air, tan δ is the loss tangent in the material, ε′ is the real part of the dielectric constant, and *f* is the frequency of application. The heating rate subsequently achieved through RF application is

2 $$ \frac{\varDelta T}{t}\left[\frac{K}{s}\right]=\frac{D_H}{C_V}, $$

where *C*_*V*_ is the volume-specific heat capacity.

To determine the heat capacity of the material, *D*_*H*_ was calculated from the measured electric field amplitude, frequency, and measured ε′ and tan δ for each material. An in-house setup with water as a benchmark was used for calorimetric measurements. Samples of varying amounts were filled into acrylic vials. The acrylic vials were experimentally verified to have negligible RF response. Samples were capped and centered between two capacitive plates connected to the LRC setup. The frequency, voltage of generated RF, spacing of the plates, and sample amounts were varied and multiple sets of $\frac{\Delta T}{t}$ data recorded, where Δ*T* is the change in sample temperature, and *t* is the time of RF application. Using the measured relative dielectric constant (*ϵ* ′ ) and loss tangent (tan *δ*) at the applied RF frequency, homogenous heating was presumed and the specific heat was calculated using

3 $$ {C}_P\left[\frac{J}{kg\cdot {}^{\circ}C}\right]=\frac{D_H}{\rho_{m\cdot}\frac{\varDelta T}{t}} $$

the *C*_*p*_ of water and was determined to be 4191 $\frac{J}{kg\bullet K}$; within its error of the standard value of 4186 $\frac{J}{kg\bullet K}\operatorname{}$ [49]. Measurements for the *C*_*p*_ of the hydrogel composites were undertaken with the hydrogel composites hydrated such that the surface of the composites pressed against the meniscus of water at the surface.

## Data Availability

Data is available upon request.

## Abbreviations

EP: Electrode polarization
KBT: KF-doped barium titanite, KF-doped barium titanite dispersed nanoparticle hydrogel composite
LCST: Lower critical solution temperature
MWS: Maxwell-Wagner-Sillars
PNIPAm: Poly-N-isopropylacrylamide
PVA: Poly(vinyl alcohol)
RF: Radio frequency
